# Investigation and Analysis of Frailty and Nutritional Status in Patients With Inflammatory Bowel Disease

**DOI:** 10.1093/crocol/otaf010

**Published:** 2025-03-24

**Authors:** Jin-Feng Liu, Qiu-Xia Jiang, Juan Liu, A-Lan Liu, Yu-Han Wang

**Affiliations:** Department of Gastroenterology, The First Affiliated Hospital of USTC (Anhui Provincial Hospital), 17 Lujiang Road, Hefei, Anhui, China; Department of Gastroenterology, The First Affiliated Hospital of USTC (Anhui Provincial Hospital), 17 Lujiang Road, Hefei, Anhui, China; Department of Gastroenterology, The First Affiliated Hospital of USTC (Anhui Provincial Hospital), 17 Lujiang Road, Hefei, Anhui, China; Department of Gastroenterology, The First Affiliated Hospital of USTC (Anhui Provincial Hospital), 17 Lujiang Road, Hefei, Anhui, China; Department of Gastroenterology, The First Affiliated Hospital of USTC (Anhui Provincial Hospital), 17 Lujiang Road, Hefei, Anhui, China

**Keywords:** inflammatory bowel disease, frailty phenotype, nutritional status

## Abstract

**Background:**

To analyze the current status of frailty and the primary factors influencing frailty in patients with inflammatory bowel disease (IBD).

**Methods:**

We conducted a study using a fixed-point consecutive sampling method to investigate hospitalized patients with IBD aged 18 years or older in the Gastroenterology Department of a general hospital in Anhui, China, from July 2022 to July 2023. We also assessed the prevalence of frailty and malnutrition using the frailty phenotype scale (trial of fatigue, grip strength, physical activity, walking speed, and weight loss) and the Global Leadership Initiative on Malnutrition criteria to analyze the factors influencing frailty.

**Results:**

A total of 300 patients with IBD were included. Of them, 21.67% were classified as frail, 46.67% were prefrail, 31.6% were nonfrail, 35% showed nutritional risk, and 33% were malnourished. The results of bivariate correlation analysis showed that frailty scores were correlated with age, white blood cell count, faecal calprotectin, and C-reactive protein levels and were negatively correlated with body mass index (BMI), hemoglobin, albumin (ALB), and pre-albumin (PALB) levels (*r* = −0.35, −0.45, −0.55, −0.44, *P* <.01). The results of multiple linear regression analysis showed that BMI scores, nutritional status, disease state, and ALB levels were important factors influencing frailty (*P* <.05).

**Conclusions:**

The patients with IBD were frail and prefrail, with a high prevalence of malnutrition. Lower BMI scores, a poor nutritional status, a worse disease state, and lower ALB levels were risk factors for frailty. A cyclical relationship was identified between frailty and malnutrition, with each condition exacerbating the other.

## Introduction

The original concept of frailty was based on the state of cardiovascular health, emphasizing the clinical syndrome of age-related decline in the reserve and function of multiple physiological systems, resulting in increased vulnerability to acute daily stress.^1^ Frailty is linked to increased vulnerability to stressors due to defects in multiple physiological systems, such as the immune, musculoskeletal, endocrine, and nervous systems.^2^ Frailty is associated with adverse outcomes in patients with various chronic diseases, including cirrhosis, heart failure, chronic kidney disease, and diabetes, as well as those who underwent surgery.^3^ Frailty was originally related to the decline in health and nutrition associated with aging, but it is now thought to be dysregulation of the body’s multisystem homeostatic reserves, and it can occur at any age.^4^

Inflammatory bowel disease (IBD) is a systemic, chronic, persistent, and relapsing disease of the intestinal flora, resulting in changes in immune function and inflammatory response.^5^ Approximately 7 million people worldwide have been diagnosed with IBD, causing severe economic stress and medical burden to families and society.^6^ Severe IBD often presents with frailty-related features, including weight loss, fatigue, and sarcopenia.^7^ Therefore, frailty may be a component of IBD.^7^ A recent study reported the prevalence of frailty among patients with IBD at 18%, and it was identified as a significant independent predictor of mortality.^8^ In a study of 135 patients diagnosed with IBD and frailty aged more than 65 years, 23% had increased frailty, and 44% had impaired mobility.^9^ A retrospective cohort study was conducted based on an electronic medical record system that used a frailty index to assess the frailty status of patients. The results showed that the mean C-reactive protein (CRP) level was higher in frail patients with IBD than in healthy patients with IBD.^10^ According to Faye et al.,^4^ the prevalence of frailty among hospitalized patients with IBD has shown a steady increase, rising from 10.2% in 2010 to 11.5%. Furthermore, after adjusting for age and comorbidities, the incidence of frailty is also found to be associated with higher rates of readmission and increased mortality. Kochar et al.^11^ conducted a study which indicated that individuals who exhibited signs of frailty prior to the administration of biologics or immunosuppressive medications faced a doubled risk of subsequent infections. One study was validated. A study using a validated frailty index in a cohort of 943 patients undergoing colectomy for UC found that frailty was an independent and significant predictor of septic and cardiopulmonary complications, severe morbidity, reoperation, and total mortality.^12^ The European Crohn’s and Colitis Organization recommends that clinicians assess patient frailty.^13^

Despite the increasing awareness of frailty, it continues to be underdiagnosed in patients with IBD. The assessment of frailty in patients with IBD can aid in the identification of those at higher risk for developing diseases or treatment-related complications. In this study, we utilized the frailty phenotype (FP)^1^ scale and the Global Leadership Initiative on Malnutrition (GLIM)^14^ consensus on malnutrition diagnostic criteria to investigate the prevalence of frailty and malnutrition in hospitalized IBD patients, as well as to analyze the correlation between frailty and nutritional status and their impact on postoperative outcomes.

## Materials and Methods

### Patients

The study was conducted in patients with IBD at a single center between July 2022 and July 2023. This study protocol was reviewed and approved by the Medical Research Ethics Committee, with approval number 2022KY-372.The inclusion criteria were a confirmed diagnosis of IBD,^15^ age of 18 years and older, and willingness to participate. Exclusion criteria: bacillary dysentery, amoebic dysentery, infectious enteritis such as chronic schistosomiasis, ischemic enteritis, radiation enteritis, and intestinal cancer; patients with osteoarthritis and other motor system diseases; patients with other autoimmune diseases; patients with severe complications including intestinal obstruction, fistula, abdominal abscess, massive bleeding of lower gastrointestinal tract, intestinal perforation, toxic megacolon, intraepithelial neoplasia, and cancer; people with mental illness; and others had various questionnaire data collection contraindications.

This study was an investigation study, and the purpose was to investigate the incidence of frailty in IBD patients. According to the results of literature research, the incidence of frailty in IBD patients was about 25%, the allowable error was 5%, and the confidence was 1 – α = 0.95. The sample size to be investigated was calculated by PASS11 software, *N* = 306. A total of 306 hospitalized patients with IBD were given questionnaires. Six questionnaire responses were excluded due to incomplete data (the effective rate was 98%). Finally, 300 patients participated in the investigation.

### Materials

A self-made questionnaire was used to collect general information and disease-related information, including age, gender, occupation, laboratory examination results, and other results, from patients with IBD. The Crohn’s Disease Activity Index was used to assess disease activity in patients with CD. Scores of <150 were defined as clinical remission, 150-220 as mild activity, 220-450 as moderate activity, and >450 as severe activity. Disease activity in patients with ulcerative colitis (UC) was assessed by the modified Mayo score, where a sum of the total scores of <2 indicates clinical remission, scores of 3-5 indicate mild activity, scores of 6-10 indicate moderate activity, and scores of 11-12 indicate severe activity.

#### Frailty phenotype

According to the definition proposed by Fried et al. in 2001,^16^ frailty includes 5 aspects that have been widely used in clinical and research applications.^17^ (1) Weight loss, and specifically unintentional weight loss, which refers to weight loss of more than 3 kg or weight loss of more than 5% of the previous year’s body weight. (2) Walking speed is assessed as the time used to walk 4.57 m. This time is measured and combined with height and gender.^17^ (3) Grip strength is measured using a handheld electronic grip meter (model: Xiangshan EH101). Measurements are taken palm-inward, dial-outward, body upright, at an upper arm and forearm angle of 90°, and the forearm parallel to the ground in accordance with the patient’s hand type-appropriate adjustment of the grip distance. The tool is not in contact with the body or clothing, and measurements are conducted 3 times. The average value is taken and combined with gender and body mass index (BMI). (iv) Physical activity is measured using the International Physical Activity Questionnaire Short Form to count the number of calories consumed in a week. Low physical activity is defined as <383 Kcals/week (metabolic equivalent of energy) for males and <270 Kcals/week (METs) for females.^18^ (v) For fatigue, 2 items from the Center for Epidemiologic Studies Depression Scale are evaluated^19^: Item 1: “I’ve had a hard time doing everything for the past week,” and item 2: “I have been unable to walk forward for the past week.” Scoring is as follows: <1 day, 0 points; 1-2 days, 1 point; 3-4 days, 2 points; and >4 days, 3 points. Scores of 2-3 points for any item indicate fatigue. A score of 3 or above indicates frailty, a score of 1-2 indicates prefrailty, and 0 indicates lack of frailty. In the present study, prefrailty and no-frailty were combined into one category of nonfrailty.

#### Nutritional risk screening and malnutrition diagnosis

The GLIM criteria for assessing malnutrition consist of 2 steps.^20^ Step 1: Nutritional risk screening is conducted within 24 hours of admission using the NRS-2002 scale, which comprises 3 parts: impaired nutritional status, disease severity, and age. A score of ≥3 indicates that the patient is at nutritional risk. A score of less than 3 indicates no present risk, and the scale should be reassessed regularly every week. Cronbach’s α coefficient and validity of the scale were found to be 0.88 and 0.78. Step 2: When the NRS 2002 score is positive, a diagnosis of malnutrition needs to be made within 48 hours. The specific content includes 2 phenotype indicators (involuntary weight loss and low BMI) and 2 etiological indicators (reduced food intake or nutrient absorption and utilization disorders and inflammation). A diagnosis of malnutrition can be made when a patient meets at least 1 phenotype indicator and 1 etiological indicator. The degree of malnutrition is graded according to the proportion of involuntary weight loss in phenotypic indicators. Moderate malnutrition is diagnosed for a weight loss between 5% and 10% in the past 6 months or between 10% and 20% in the past 6 months. Severe malnutrition is diagnosed for a weight loss of greater than 10% in the past 6 months or over 20% in a month.

#### Laboratory tests

Fasting blood tests for hemoglobin (Hb), white blood cell (WBC) count, serum albumin (ALB), serum pre-albumin (PALB), serum total protein (TP), and high sensitivity C‐reactive protein (CRP) were collected from the enrolled patients early in the morning on day 2 of hospitalization.

### Design and Data Collection

A fixed-point consecutive sampling method was utilized, in which participants were recruited from a tertiary hospital in central-eastern China. Data were gathered through field surveys conducted between 2022 and 2023. Three study nurses recruited potential participants from the inpatient ward within the first week of their hospitalization.

### Statistical Analysis

Epi Data 3.1 software was used to build a database of the general information and measurement metrics for all subjects. SPSS 23.0 statistical software was used for statistical analyses. Normally distributed data are expressed as $\bar{x}$ ± s, and analysis of variance was used to compare groups. Count data are presented as case numbers and percentages. The Chi-squared test was used for comparisons between groups. A Chi-squared trend test was used to compare counts. Nonnormally distributed data are represented by median (1/4, 3/4). The Kruskal–Wallis test was used for comparisons between groups. Multifactorial logistic regression was employed to examine the association between disease severity, frailty, and malnutrition. All tests were conducted with a two-sided approach, and statistical significance was defined as *P* <.05.

## Results

### General Characteristics of Patients With IBD

**Figure 1.**
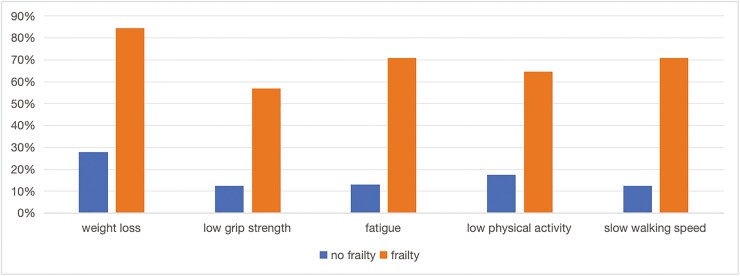
Percentage of nonfrail and frail individuals for each FP criterion.

Table 1 presents the characteristics of the 300 patients included in the final analysis of the FP questionnaire responses. The majority of the participants were male (63.33%, *n* = 190), aged 18-77 years, with a mean age of 37.24 ± 14.06 years. Two hundred and twelve patients (70.67%) had CD, and 88 patients with UC (29.33%) experienced disease duration ranging from 1 to 40 years. Sixty-five cases were classified into the frail group (21.67%), 140 cases into the prefrail group (46.67%), and 95 cases into the nonfrail group (31.66%), with the most relevant frailty criterion in frail individuals with weight loss (85%), followed by fatigue and slow walking speed (71%). Figure 1 depicts the distribution of the percentage of frail and prefrail individuals according to each FP criterion. One hundred and five cases were considered to be at nutritional risk (35%), 99 had malnutrition (33%), and 17.3% of the cases had a plasma ALB level lower than the normal value. The median age of the patients in the frail group was 43 years. The proportions of nutritional risk and malnutrition in frail, prefrail, and nonfrail patients were 48.50% and 47.60%, 37.10% and 35.40%, and 15.20% and 16.20%, respectively. These 2 indexes were higher in frail patients than in prefrail and nonfrail patients, and the differences were statistically significant (*P* <.05). Statistically significant differences were observed between the disease stage of the patients and their frailty state, gender, BMI, and laboratory indicators (*P* <.05). A subset of the patients included in this study was in clinical remission of their disease. These patients required periodic hospitalization with biologic therapy to sustain remission and were included in the study. The comparison of patient FPs is shown in Table 1.

**Table 1.** Participant characteristics and univariate analysis (*n *= 300).

Variables	Frailty status			*P*
	Normal (*n* = 95)	Prefrail (*n* = 140)	Frail (*n* = 65)	
Age (year) *M*(*P*_*25,*_,*P*_*75*_)	32.0 (26.0, 43.0)	33.0 (26.0, 46.0)^a^	43.0 (28.5, 57.0)^a^^,^^b^	.00
Gender *n* (%)				.09
Male	72 (37.90)	86 (45.30)^b^	32 (16.80)^a^^,^^b^	
Female	23 (20.90)	54 (49.10)^b^	33 (30.00)^a^^,^^b^	
Duration of disease (years) *M*(*P*_*25*_*,P*_*75*_)	3.0 (2.0,6.0)	3.0 (1.0, 6.0)^a^	1.5 (1.0, 5.0)^a^^,^^b^	.01
Marital status *n*(%)				.29
Married	53 (29.80)	81 (45.50)	44 (24.70)	
Other (single, divorced, unknown)	42 (34.40)	59 (48.40)	21 (17.20)	
IBD phenotype *n*(%)				<.01
CD	79 (37.30)	98 (46.20)	35 (16.50)^a^^,^^b^	
UC	16 (18.20)	42 (47.70)	30 (34.10)^a^^,^^b^	
With history of IBD surgery *n*(%)				.03
No	78 (80.90)	126 (88.14)	62 (94.74)^a^^,^^b^	
Yes	17 (19.10)	14 (11.86)	3 (5.26)^a^^,^^b^	
Disease state *n*(%)				<.01
Remission	79 (50.30)	74 (47.10)	4 (2.50)^a^^,^^b^	
Mild active	12 (21.80)^a^	28 (50.90)^a^	15 (27.30)^a^	
Moderately active	4 (6.10)	30 (45.50)	32 (48.50)^a^^,^^b^	
Severe activity	0 (0.00)	8 (36.40)	14 (63.60)^a^^,^^b^	
BMI(kg/m^2^)$\bar{x}$ ± s	21.60 ± 3.27	21.06 ± 2.77^a^	18.71 ± 3.46^a^^,^^b^	<.01
Hb (g/L) *M*(*P*_*25*_*,P*_*75*_)	133.00 (118.00, 144.00)	127.00 (111.25, 139.00)^a^	109.00 (93.00, 118.00)^a^^,^^b^	<.01
WBC(*10^9^/L)$\bar{x}$ ± s	5.47 ± 1.44	5.95 ± 2.28^a^	6.38 ± 2.36^a^^,^^b^	.04
ALB(g/L)$\bar{x}$ ± s	41.25 ± 3.05	39.88 ± 4.20^a^	34.41 ± 5.64^a^^,^^b^	<.01
PALB (mg/L)$\bar{x}$ ± s	233.54 ± 41.72	223.16 ± 60.00^a^	166.22 ± 64.04^a^^,^^b^	<.01
Faecal Calprotectin(ug/g) *M*(*P*_*25*_*,P*_*75*_)	62.00 (22.00, 195.00)	100.50 (50.75, 279.75)^a^	153.00 (54.00, 349.50)^a^^,^^b^	.00
CRP(mg/L) *M*(*P*_*25*_*,P*_*75*_)	3.30 (3.30, 5.00)	3.41 (3.20, 10.78)^a^	14.50 (3.36, 47.45)^a^^,^^b^	<.01
GLIM Diagnosing malnutrition, n(%)	16 (16.20)	35 (35.40)^a^	48 (48.50)^a^^,^^b^	<.01
GLIM Diagnosing nonmalnutrition, *n*(%)	79 (39.30)	105 (52.20)^a^	17 (8.50)^a^^,^^b^	
Biological agents using *n*(%)				<.01
No	12 (13.60)	43 (48.90)	33 (37.50)^a^^,^^b^	
Yes	83 (39.20)	97 (45.80)	32 (15.10)^a^^,^^b^	
NRS-2002* ≥ *3 *n*(%)	16 (15.20)	39 (37.10)^a^	50 (47.60)^a^^,^^b^	<.01
NRS-2002* < *3 *n*(%)	79 (40.50)	101 (51.80)^a^	15 (7.70)^a^^,^^b^	

Chi-squared and *P*-values were calculated by comparing the data of the frailty group, prefrailty group, and nonfrailty group.

Abbreviations: ALB, serum albumin; BMI, body mass index; CD, Crohn’s disease; CRP, C‐reactive protein; GLIM, Global Leadership Initiative on Malnutrition; Hb, hemoglobin; IBD, Inflammatory Bowel Disease; NRS-2002, Nutritional Risk Screening Score 2002; PALB, serum pre-albumin; UC, Ulcerative Colitis; WBC, white blood cell count.

^a^ *P* <.05 compared with the nonfrailty group.

^b^ *P* <.05 compared with the prefrailty group.

### Factors Influencing Frailty in IBD Patients and the Association Between Frailty and Malnutrition

The general data of 300 patients were analyzed by univariate analysis. The results indicated no statistically significant differences in patients’ sex, duration of disease, or marital status (*P* >.05). However, statistically significant differences were seen in frailty scores among age, GLIM, IBD phenotype, disease state, and whether biological agents were being used (*P* <.05) (Table 2).

**Table 2.** Correlation analysis of frailty assessment scores and general information.

Item	Count	Frailty phenotype scale,$\bar{x}$ ± s	*t/F*	*P*
Gender				
Male	190	1.48 ± 1.26	4.00	.16
Female	110	1.80 ± 1.36		
Marital status				
Others	122	1.21 ± 1.19	2.17	.06
Married	178	1.54 ± 1.40		
Disease state				
Remission	157	0.70 ± 0.82	58.55	<.01
Mild active	55	1.65 ± 1.17		
Moderately active	66	2.35 ± 1.32		
Severe activity	22	3.00 ± 1.35		
GLIM				
Nonmalnutrition	201	0.97 ± 0.97	7.98	<.01
Malnutrition	99	2.29 ± 1.50		
IBD phenotype				
CD	212	1.24 ± 1.29	12.44	<.01
UC	88	1.82 ± 1.34		
Biological agents using				
No	88	2.07 ± 1.40	34.41	.03
Yes	212	1.13 ± 1.19		

Abbreviations: Frailty, loss of weight, fatigue, and decreased grip strength, physical activity, and walking speed; GLIM, Global Leadership Initiative on Malnutrition. IBD, inflammatory bowel disease; UC, ulcerative colitis; CD, Crohn’s disease.

The overall frailty score of all 300 patients was (1.41 ± 1.33). The results of bivariate correlation analysis showed that frailty scores were correlated with age, WBC count, faecal calprotectin, and CRP levels and were negatively correlated with BMI, Hb, ALB, and PALB levels (*r* = −0.35, −0.45, −0.55, −0.44, *P* <.01) (Table 3).

**Table 3.** Correlation analysis of frailty scores with age, BMI, duration of disease, and laboratory indicators (CRP, Hb, ALB, PALB, WBC, and faecal calprotectin).

Item	Frailty phenotype scale,$\bar{x}$ ± s	
	*r*	*P*
Age	0.29	<.01
BMI	-0.35	<.01
Duration of disease	-0.05	.367
Hb	-0.45	<.01
WBC	0.13	.03
ALB	-0.55	<.01
PALB	-0.44	<.01
Faecal Calprotectin	0.12	.04
CRP	0.22	<.01

Abbreviations: ALB, serum albumin; BMI, body mass index; CRP, C-reactive protein; Frailty, loss of weight, fatigue, and decreased grip strength, physical activity, and walking speed; Hb, hemoglobin; PALB, serum pre-albumin; WBC, white blood cell count.

The results of multiple linear regression analysis showed that BMI scores (*P* =.011, 95% CI: −0.097 to −0.013), nutritional status (*P* =.003, 95% CI: 0.154-0.761), disease state (*P* <.001, 95% CI: 0.298-0.636), and ALB levels (*P* <.001, 95% CI: −0.100 to −0.037) were important factors influencing frailty (*P* <.05) (Table 4).

**Table 4.** Multivariate linear regression analysis of frailty assessment scores.

Item	Unstandardized regression coefficients		Standardized regression coefficients			95% CI	
	*β*	SE	*β’*	*t*	*P*	Lower bound	Upper bound
Age	0.009	0.005	0.096	1.952	.052	0.000	0.018
BMI	−0.055	0.021	−0.135	−2.559	.011	−0.097	−0.013
IBD phenotype	−0.134	0.157	−0.046	−0.851	.396	−0.443	0.176
Biological agents using	−0.231	0.145	−0.080	−1.596	.111	−0.517	0.054
GLIM	0.458	0.154	0.162	2.969	.003	0.154	0.761
Disease state	0.467	0.086	0.355	5.451	<.001	0.298	0.636
Hb	−0.002	0.003	−0.026	−0.446	.656	−0.008	0.005
WBC	0.016	0.029	0.026	0.567	.571	−0.040	0.073
CRP	0.002	0.002	0.040	0.892	.373	−0.002	0.005
ALB	−0.068	0.016	−0.254	−4.270	<.001	−0.100	−0.037
PALB	0.001	0.001	0.068	1.141	.255	−0.001	0.004
Faecal Calprotectin	0.000	0.000	0.027	0.617	.538	0.000	0.001
(Constant)	4.387	0.816	-	5.379	<.001	2.781	5.922

Abbreviations: ALB, serum albumin; BMI, body mass index; CRP, C-reactive protein; GLIM, Global Leadership Initiative on Malnutrition; Hb, hemoglobin; PALB, serum pre-albumin; WBC, white blood cell count.

## Discussion

The prevalence of frailty was high in IBD patients. This study revealed that among 300 patients, frailty and prefrailty states were detected in 21.67% and 46.67%. These rates are consistent with the reported detection rates of frailty in IBD patients (10.6%-53.3%) in both domestic and international literature and higher than those observed in the general population aged 65 years or older.^21–23^ The findings of this study also revealed that 35% of the patients were at risk for malnutrition, and 33% were diagnosed with malnutrition. These results indicate a poor nutritional status among individuals with IBD.^24^ Frailty is a clinical syndrome in which the body’s vulnerability increases and its antistress ability decreases due to the reduction in physiological reserves and the occurrence of multisystem disorders. Minor external stimuli can lead to adverse events, which was first proposed by Fried et al. in 2001.^16^ Frailty is correlated with multiple adverse consequences in IBD patients, such as elevated mortality, heightened risk of infection, and increased CRP levels.^10,11,21^ However, frailty and inflammation are dynamically interrelated, and interventions aimed at the factors influencing frailty can ameliorate the progression of inflammation and thereby control the recurrence of the disease.^10^ Thus, the early identification of factors influencing frailty is particularly crucial.

This study showed that nutritional status, rather than age, plays a crucial role in the development of frailty in patients with IBD. Nutrition plays a crucial role in controlling IBD and its associated vulnerable phenotypes. As an important disease control indicator, malnutrition is a common problem in patients with IBD, with an incidence of about 49.5%.^25^ It is mainly characterized by protein-caloric malnutrition. This study showed that 35% of the patients had an NRS-2002 score of more than 3 points, and 17.3% had a plasma ALB level lower than the normal value. Although age was considerably different in the univariate analysis of patients with IBD, this correlation was not seen in the multivariate analysis after combining other factors. One possible cause of malnutrition and frailty in patients with IBD is the presence of inflammation, which impairs intestinal absorption function and limits the intake and absorption of nutrients. Patients with IBD also often have loss of appetite, diarrhea, and other symptoms, which affect the intake and absorption of nutrients, leading to muscle atrophy, weight loss, and overall weakness. Malnutrition and frailty are correlated in patients with IBD. Frailty can further increase the level of malnutrition, creating a vicious cycle. Therefore, patients with IBD should maintain a good nutritional state through a reasonable diet and nutritional supplements to prevent or improve malnutrition and frailty. Regular nutritional assessment and monitoring and the timely detection and intervention of malnutrition and frailty are also important measures for managing patients with IBD.

The findings of this study indicate that frailty is correlated with disease state, which aligns with the conclusions drawn by Cong et al.^26^ Disease activity refers to the severity of a disease and the activity of inflammation. Patients with high levels of inflammatory activity tend to have more severe symptoms, such as diarrhea, abdominal pain, and anemia. These symptoms lead to the deterioration of the patient’s physical condition and promote changes in their body composition. Body composition indices, such as Hb, ALB, the subcutaneous fat index, visceral fat index, and muscle content index in patients with active CD, are lower than those in patients in remission.^27^ Furthermore, disease activity in patients with IBD can lead to nutritional issues. Patients in the active stage of the disease not only experience difficulties with nutrient absorption but also typically have decreased appetite, which exacerbates the occurrence of malnutrition and weakness.^28^ Disease activity also impacts the mental state of the patient. Both mood disorders and cognitive performance in IBD patients over time are linked to disease activity, which is primarily characterized by fatigue and increased disease burden.^29,30^ These data reflect the important role of disease activity in frailty in IBD patients, as well as the dynamic nature of frailty. The degree of frailty varies over time, depending on the presence or absence of risk factors. Therefore, controlling the disease activity of patients is crucial to prevent and manage nutritional and frailty issues in IBD patients. The effective management of patient frailty can also serve as a significant prognostic factor in the risk stratification of IBD patients and help guide the selection of the most appropriate treatment plan. Despite the absence of statistical significance, the study has revealed that a substantial proportion of patients in remission, amounting to 47%, displayed signs of prefrailty. This finding has elicited a significant question: should the assessment of frailty be incorporated into the comprehensive evaluation of patients to ensure the provision of more appropriate diagnostic and therapeutic interventions? Further clinical research is deemed essential to explore this issue in depth.

Research has indicated that the prevalence of frailty in patients with CD and UC differs due to their distinct disease behaviors and characteristics, as well as the potential impact of these characteristics on the development of frailty.^30^ Evidence suggests that individuals with CD are at a higher risk for developing frailty.^21^ However, this was not found in the present study, possibly due to differences in the measurement tools and sample size utilized.

### Limitations

This study had some limitations. First, the questionnaire was self-reported and had limited reliability for the true condition of the patient. Second, the sample sources in this study were obtained by convenient sampling. While a certain amount of data can be collected quickly, the results may not be comprehensive and representative. Therefore, longitudinal studies are needed at a later stage to determine the causal relationship between the variables. Third, data were collected from a general hospital in central-eastern China and may not reflect the general situation of patients with IBD. Therefore, future studies should conduct longitudinal studies on a larger sample size from a larger area to more precisely reflect the frailty of patients with IBD and provide a reference and basis for clinical treatment and care.

## Conclusion

Individuals with IBD are at a higher risk of developing frailty, which is closely associated with plasma ALB levels, nutritional status, and disease activity. Actively promoting disease remission among patients, conducting thorough nutritional assessments, and addressing any malnutrition present are crucial in clinical practice. The study findings indicate the importance of the early identification of frailty in IBD patients and the implementation of appropriate interventions to significantly improve prognosis and reduce disability. Preplanned exercise interventions aimed at increasing muscle strength and mass, as well as enhancing physical activity, should be considered. Additionally, psychological interventions and measures to alleviate fatigue, prevent and reduce frailty, and lessen the burden of disease should be administered. The ultimate goal in treating chronic inflammatory diseases is to restore normal functional status; however, it is important to recognize that frailty is a dynamic concept influenced by various factors that may change over time. Therefore, further research is necessary to understand the dynamic trajectory of frailty to enhance disease remission and minimize adverse events in IBD patients.

## Data Availability

All relevant data are within the manuscript and its Additional files.
